# Clinical Lectures on Diseases of the Nervous System

**Published:** 1874-12

**Authors:** 


*Clinical Lectures on Diseases of the Nervous System.* By William A. Hammond, M. D. Reported and Edited by T. M. B. Cross, M. D. New York: D. Appleton & Co., 1874. Buffalo: Martin Taylor.

The twenty lectures comprising this volume, were delivered at the New York State Hospital for Diseases of the Nervous System, and at the Bellevue Hospital Medical College. Dr. Cross, the editor, has taken pains to report the lectures in as full a manner as possible, and has also carefully written the history of each case. He informs his readers that he does not claim for the work a place as an exhaustive treatise upon Diseases of the Nervous System, but that it is intended as an addition to the clinical literature of nervous diseases. The lectures are upon the following topics: Partical cerebral anæmia from thrombosis and embolism, alternate or cross hemiplegia, congestion and chronic inflammation of the spinal cord, reflex paralysis, lead paralysis, facial paralysis, cerebral hemorrhage, posterior spinal sclerosis, convulsive tremor, epilepsy, sciatica, organic infantile paralysis, etc., etc.

There are several topics which we have not named, but the more important ones have been mentioned. The lectures are well edited, and the reports of cases are well made. The views advanced are essentially those contained in Dr. Hammond's work, on Diseases of the Nervous System, with which many of our readers are doubtless familiar.

